# Cholecystoenteric Fistula: A Single-Center Experience of Seven Cases with Unusual Complications of Gallstone Disease

**DOI:** 10.7759/cureus.81350

**Published:** 2025-03-28

**Authors:** Karamveer Singh, Nayana S Kumar, Manoj Joshua Lokavarapu, Satish Ammapalem, Monisha Selvarasu, Amit Gupta

**Affiliations:** 1 Department of General Surgery and Division of Organ Transplant, All India Institute of Medical Sciences, Rishikesh, Rishikesh, IND; 2 Department of General Surgery, All India Institute of Medical Sciences, Rishikesh, Rishikesh, IND

**Keywords:** biliary enteric fistula, biliary fistula, cholecystoduodenal fistula, cholecystoenteric fistula, cholelithiasis, gallstone disease, hepatobililary surgery, intraoperative diagnosis, laparoscopic cholecystectomy

## Abstract

Background: Cholecystoenteric fistulas (CEFs) are rare complications of chronic calculous cholecystitis, often diagnosed intraoperatively due to their nonspecific clinical presentation and challenges in preoperative detection. This study analyzes the surgical management and outcomes of CEFs at a tertiary care center.

Methods: A retrospective analysis was conducted on all patients who underwent surgery for CEF in the hepatopancreaticobiliary unit of the Department of Surgery at the All India Institute of Medical Sciences, Rishikesh, India, between June and December 2024. Data on preoperative characteristics, biochemical parameters, intraoperative findings, surgical techniques, conversion rates, and postoperative outcomes were collected and analyzed.

Results: Seven patients were included, with a mean age of 55.6 years (range: 49-69 years). Abdominal pain was the most common symptom (seven, 100%), while cholangitis was present in three (42.86%) cases. Gallstones were found in all patients (seven, 100%) and bile duct stones in two (28.57%). Multiple fistulas were observed in four (57.14%) cases, with cholecystocolonic fistulas (CCFs) and cholecystoduodenal fistulas (CDFs) being the most common combinations. Laparoscopic surgery was attempted in four (57.14%) cases but required conversion to open surgery. Bilioenteric anastomosis with Roux-en-Y hepaticojejunostomy was performed in three (42.86%). Postoperative complications included surgical site infections in two (28.57%) of the cases, and one (14.29%) died in the postoperative period. The mean hospital stay was 13.4 ± 3.8 days.

Conclusion: Cholecystoenteric fistula remains a diagnostic and surgical challenge due to its nonspecific presentation and intraoperative detection. While preoperative imaging can aid in diagnosis, most cases are identified intraoperatively, necessitating modifications in the surgical approach. Advances in laparoscopic techniques have reduced conversion rates, but complex cases still require open surgery. Further studies with larger cohorts are needed to refine diagnostic and therapeutic strategies.

## Introduction

Cholecystoenteric fistula (CEF) was first documented by Bartholin in 1654; it’s an uncommon consequence of cholelithiasis, having an incidence of 3% to 5% within patients having cholelithiasis as well as 0.15% to 4.8% within those following biliary surgery [1-4]. It is characterized by a spontaneous, pathological communication between the gallbladder and the gastrointestinal tract, most often secondary to chronic inflammation caused by gallstones. These abnormal connections, collectively referred to as CEFs, include cholecystogastric fistulas (CGF), cholecystoduodenal fistulas (CDF), and cholecystocolonic fistulas (CCF). The duodenum is the most common site, comprising 75% to 80% of all such fistulas, followed by the colon and stomach [5]. An inflamed, constricted gallbladder adherent to adjacent viscera with a history of chronic calculous cholecystitis should alert the surgeon regarding the CEF.

The preoperative diagnosis of CEF has been challenging due to the vague nature of its symptoms, which include flatulence, dyspepsia, vomiting, diarrhea, and abdominal pain [6]. This poses a challenge to accurate preoperative diagnosis. Biliary fistulas are categorized as external or internal. External biliary fistulas (EBFs) are generally of iatrogenic origin. An internal biliary fistula (IBF) represents an abnormal connection between the biliary system and internal organs. It is categorized into bronchobiliary, biliobilary, as well as bilioenteric [7]. Chronic calculous cholecystitis is the most common etiology of IBF; less prevalent causes encompass peptic ulcer disease along with neoplasms of the pancreas, stomach, and colon, as well as the duodenum [7-9].

## Materials and methods

Study design and setting

This retrospective study was conducted in the hepatopancreaticobiliary unit of the Department of Surgery at the All India Institute of Medical Sciences, Rishikesh, India. We analyzed all patients who were evaluated for symptomatic gallstone disease and were intraoperatively found to have CEF, undergoing surgical intervention between June 2024 and December 2024.

Patient selection

All patients who were diagnosed intraoperatively with CEF during surgery for symptomatic gallstone disease in our unit during the study period were included. Patients were identified retrospectively from hospital records and operative logs. No preoperative imaging criteria were used for diagnosis, as the condition was confirmed intraoperatively.

Inclusion and exclusion criteria

We included patients who were 18 years of age or older and were found to have a CEF during surgery. Only those who underwent surgical intervention for CEF within our unit between June 2024 and December 2024 were considered for inclusion. No specific exclusion criteria were applied in this study.

Data collection

The Institutional Ethics Committee of All India Institute of Medical Sciences, Rishikesh, approved (AIIMS/IEC/25/125) the study before data collection. Patient data were retrieved from medical records, operative notes, and electronic hospital databases. The following variables were collected and analyzed.

Preoperative Characteristics

These included demographics (age, gender), presenting symptoms, comorbidities, prior history of gallstone disease, and any preoperative imaging findings.

Biochemical Parameters

Liver function tests, complete blood counts, and renal function tests were the biochemical parameters included.

Intraoperative Findings

These included the presence of CEF, type of CEF, and additional biliary or gastrointestinal pathology.

Surgical Details

The type of surgical approach (laparoscopic vs. open) and procedures done were included.

Postoperative Outcomes

These included duration of hospital stay, postoperative complications (e.g., bile leak, surgical site infection, ileus), reoperation rates, and in-hospital mortality.

Statistical analysis

Descriptive statistics were used to summarize patient demographics, intraoperative findings, and postoperative outcomes. Categorical variables were presented as frequencies and percentages, while continuous variables were summarized as mean ± standard deviation (SD) or median with interquartile range (IQR), as appropriate.

## Results

**Figure 1:**
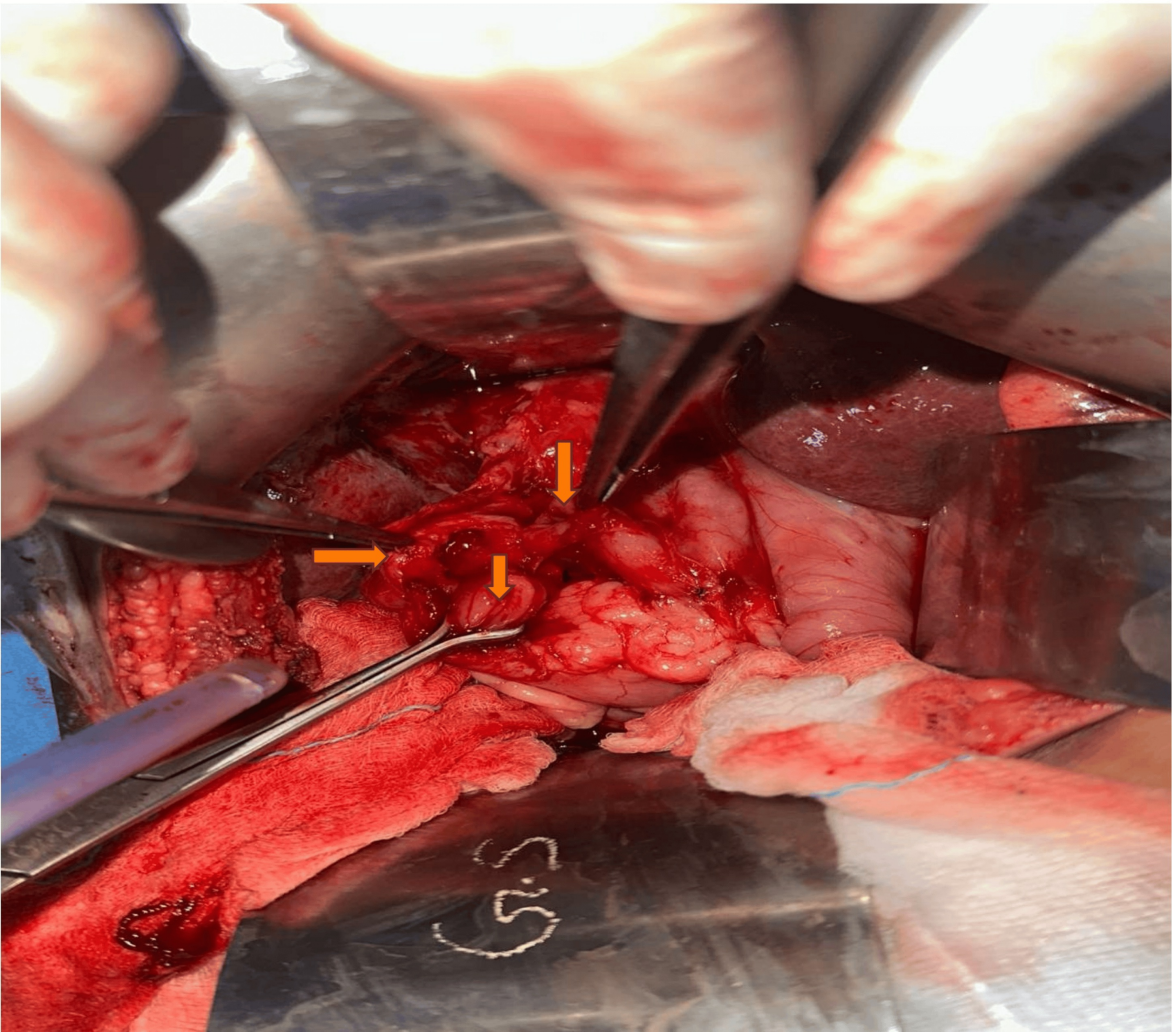
Intraoperative picture of patient no. 2 showing cholecystocolonic and cholecystogastric fistulas Image credits: Nayana S Kumar

**Figure 2:**
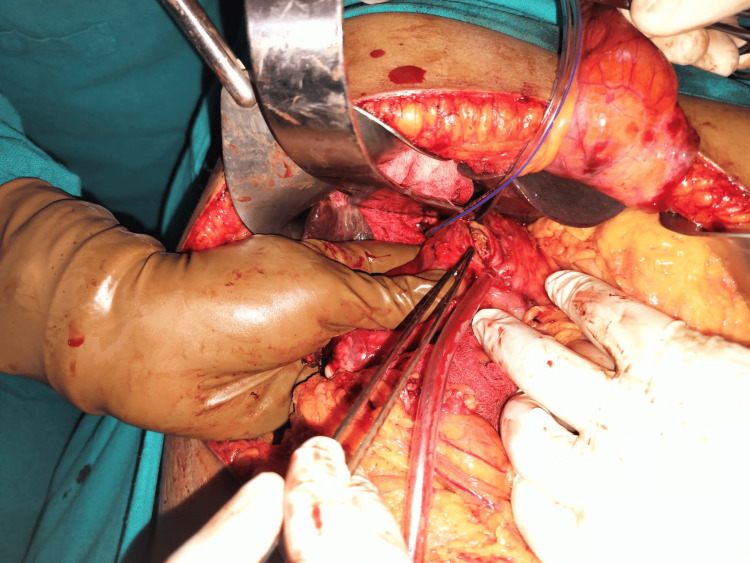
Intraoperative picture of a cholecystoduodenal fistula in patient no. 3. Image credits: Nayana S Kumar

**Figure 3:**
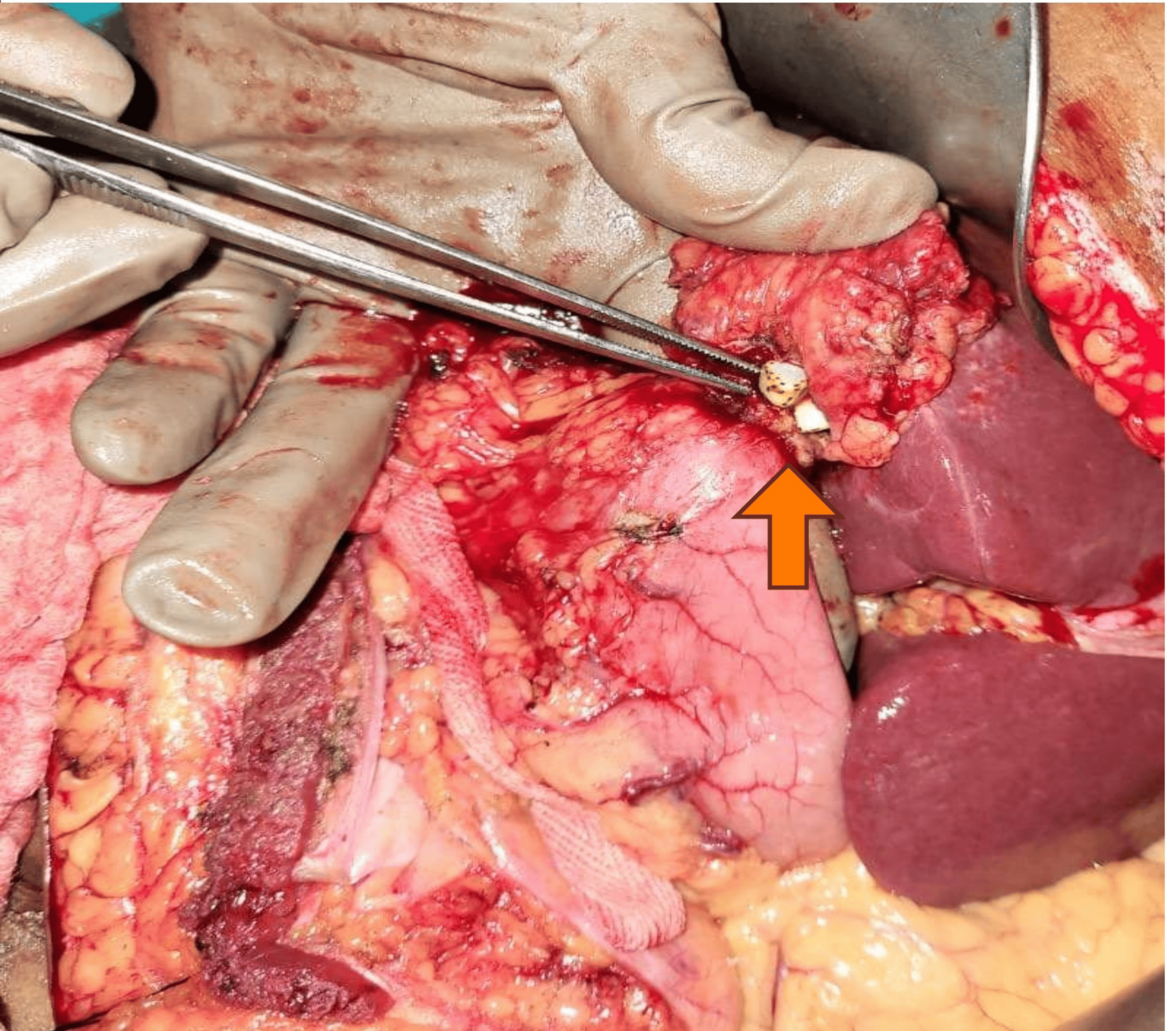
Cholecystogastric fistula in patient no. 4 (arrow) Image credits: Nayana S kumar

**Figure 4:**
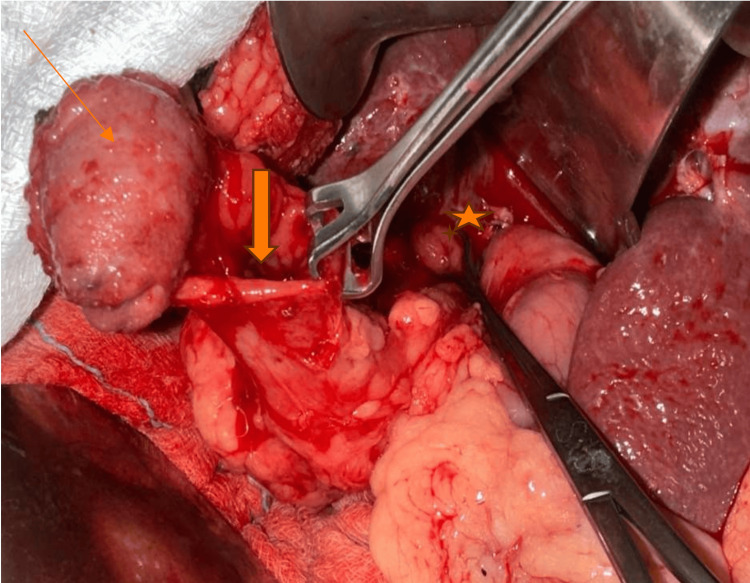
Intraoperative picture of patient no. 5 showing gallbladder wall in continuity with the colon (arrow) and an excised cholecystoduodenal fistula (star). Image credits: Nayana S Kumar

Seven patients were included in the study. Table 1 summarizes their preoperative characteristics. The mean age was 55.6 years (range: 49-69 years) with a male to female ratio of 3:4. Abdominal pain was present in all patients. Cholangitis was observed in three (42.86%), while four (57.14%) had no symptoms of cholangitis. Diabetes was present in one (14.29%) patient. Among those with cholangitis, three (42.86%) had an elevated total leukocyte count, of which one (14.29%) had an elevated total leukocyte count and abnormal liver function tests. All patients had elevated alkaline phosphatase levels. Gallstones were present in all patients, while bile duct stones were found in two (28.57%). Three (42.86%) patients underwent bilioenteric anastomosis using Roux-en-Y hepaticojejunostomy. Four (57.14%) patients had multiple fistulas: three (42.86%) had concomitant CCFs and CDFs, while one (14.29%) had both a CCF and a cholecystogastric fistula (CGF) (Figures 1-4). The mean hospital stay was 13.4 ± 3.8 days. Surgical site infections occurred in two (28.57%) patients, and one (14.29%) patient died in the postoperative period. The intraoperative findings and the procedures performed are detailed in Table 2. 

**Table 1:** Preoperative characteristics of patient with cholecystoenteric fistula M: male; F: female; DM: diabetes mellitus; SGOT: serum glutamic oxaloacetic transaminase; SGPT: serum glutamic pyruvic transaminase; HCV: hepatitis C virus

Characters	Patient 1	Patient 2	Patient 3	Patient 4	Patient 5	Patient 6	Patient7
Age (years) and sex	49/M	55/F	50/F	69/M	55/F	67/M	52/F
Symptoms	Abdominal pain, fever	Abdominal pain	Abdominal pain	Pain in the right upper abdomen	Abdominal pain	Abdominal pain, fever	Abdominal pain
Signs	Right hypochondrial tenderness	Right hypochondrial tenderness	Jaundice	Nil	Nil	Nil	Right hypochondrial tenderness
Comorbidities	Nil	Nil	DM	Nil	Nil	Nil	HCV positive
Total count (normal range: 4,000-11,000 cells/ µL)	14,100	8,420	14,530	7,730	6,390	11,800	17,040
Liver function test	-	-	-	-	-	-	-
Total bilirubin (normal range: 0.3-1.2 mg/dl)	1.6	0.84	12.9	0.98	0.39	1.09	2.73
Direct bilirubin (normal range: 0-0.2 mg/dl)	0.84	0.20	7.08	0.1	0.16	0.65	1.1
SGOT (normal range: 0-35 U/L)	50	28	53	31	32	62	58
SGPT(normal tange: 0-35 U/L)	64	31	59	50	33	77	55
Alkaline phosphatase (normal range: 30-120 U/L)	275	163	390	139	181	189	433

**Table 2:** Intraoperative findings and the surgeries done M: male' F: female; CCF: cholecystocolonic fistula; CDF: cholecystoduodenal fistula; CGF: cholecystogastric fistula; CBD: common bile duct; CBF: cholecystobiliary fistula; RYHJHJ- Roux-en-Y hepaticojejunostomy; SSI: surgical site infection; MODS: multiple organ dysfunction syndrome

No.	Age (years)/sex	Preoperative diagnosis	Intra-op findings	Surgery	Duration of hospital stay	Postoperative complications
1	49/M	Chronic calculous cholecystitis with choledocholithiasis	Dense adhesions, CDF, CCF, 3x3 cm impacted CBD stone with distal CBD stricture	Cholecystectomy + primary repair of CDF and CCF + RYHJ	16 days	SSI
2	55/F	Chronic calculous cholecystitis	Dense adhesions CGF, CCF	Cholecystectomy + primary repair of CGF and CCF	18 days	SSI
3	50/F	Chronic calculous cholecystitis with Mirizzi Type 5	Dense adhesions, CDF, hard 3x2 cm impacted CBD stone with the destruction of CBD wall (CBF)	Cholecystectomy + primary repair of CDF + RYHJ	14 days	Nil
4	69/M	Chronic calculus cholecystitis	Dense adhesion, CGF	Cholecystectomy + having excision of CGF + primary repair of pylorus	10 days	Nil
5	55/F	Chronic calculus cholecystitis	Dense adhesions, CDF, CCF	Cholecystectomy + primary repair of CDF with omental patch and CCF primary repair	12 days	Nil
6	67/M	Chronic calculous cholecystitis with Mirizzi Type 5	Dense adhesions CDF with CBF	Cholecystectomy + primary repair of CDF + RYHJ	8 days	Nil
7	52/F	Chronic calculous cholecystitis	Gall bladder contracted and hard, dense adhesions, CDF, CCF	Cholecystectomy + primary repair of CDF and CCF	16 days	MODS, death

Exemplary case presentations

Patient No. 2

A 55-year-old female patient presented with complaints of pain in her abdomen for the past six months. She underwent endoscopic retrograde cholangiopancreatography (ERCP) four months back for choledocholithiasis, and stenting was done, but no internal fistula opening was identified in ERCP. Other than pain, the patient had no other complaints. She was admitted for laparoscopic cholecystectomy and intraoperatively found to have dense adhesions, and both CCF and CGF. She underwent open cholecystectomy with primary repair of CCF and CGF. She developed a surgical site infection on postoperative day six, which was managed with IV antibiotics. The patient was discharged on postoperative day 18 with no other complications. During follow-up after one month, the patient was healthy with no complaints, and the wound fully healed.

Patient No. 3

A 50-year-old female patient presented with a history of pain in the abdomen, itching, and jaundice for one month. She was apparently asymptomatic before that. On examination, she was jaundiced and had scratch marks all over the body; investigation revealed slightly raised total counts, raised total bilirubin, and alkaline phosphatase. Intraoperatively found to have dense adhesions, CDF, a 3x2 cm hard impacted common bile duct (CBD) stone with destruction of the CBD wall and cholecystobiliary fistula (CBF) was noted. She underwent open cholecystectomy and primary repair of the CDF with Roux-en-Y hepaticojejunostomy. Her postoperative period was uneventful, and she was discharged on postoperative day 14 after one month; the patient was healthy with no complaints.

## Discussion

A CEF is uncommon and typically observed in elderly female patients. Our case series included four female and three male patients. Spontaneous IBFs usually result from chronic calculous cholecystitis. Recurrent inflammatory episodes, often accompanied by gangrenous changes in the adjacent organ and gallbladder walls, can lead to erosion and fistula formation. The incidence of IBFs is as follows: cholecystoduodenal (70%), cholecystocolonic (14%), and cholecystogastric (6%), with simultaneous occurrences being much rarer [6, 10-12].

In our case study, four patients had more than one fistula; three patients had concomitant CCFs and CDFs, while one patient had CCFs as well as CGFs (Figures 1-4). All our patients had non-specific signs or symptoms indicative of biliary fistula. Consequently, a reliable preoperative diagnosis had not been established in any of the cases. The triad of symptoms, like right hypochondrial pain, diarrhea, and cholangitis, is typically regarded as the standard medical presentation of CEF. Diarrhea is the predominant symptom in nonemergency-onset CEF because of the laxative consequences of bile acids, which bypass the distal ileum and reach unabsorbed in the transverse colon [13,14].

Symptoms of CEF, including abdominal pain, nausea, weight loss, fever, vomiting, diarrhea, flatulence, and fat intolerance, aren't specific and common to several gastrointestinal disorders, leading to a frequent lack of prior suspicion for the diagnosis. Preoperative diagnosis based on signs was unattainable in all our cases. Jaundice secondary to CBD obstruction is frequently observed in patients with cholecystocholedochal fistula (CCDF) [10,15]. In our case series, two patients had jaundice. Although two of our patients underwent ERCP for choledocholithiasis, no fistulous opening had been observed.

Literature mentions that a CT scan is the most appropriate imaging modality for the diagnosis of CEF, as sonographic evaluation is frequently challenging. A nonoperative approach can be tried in asymptomatic patients or suboptimal surgical candidates [16]. In our study, all fistulas were identified only during the surgery, as preoperatively, patients were evaluated for symptomatic cholelithiasis, and only abdominal sonography was done. One should remain vigilant for the possibility of CEF, particularly in difficult dissection scenarios during cholecystectomy involving a small, contracted, chronically inflamed, and densely adherent gallbladder, while also taking care to avert iatrogenic damage. Furthermore, intraoperative cholangiography via the gallbladder assists in detecting any pre-existing fistulous tracts. The conventional management of CEF involves cholecystectomy as well as the closure of the fistulous orifice; this approach has been uniformly applied in all the surgeries done in our series. Exploration of the CBD, tube duodenostomy, T tube insertion, choledochoduodenostomy, enterolithotomy, and hepaticojejunostomy have been utilized as adjunctive procedures when warranted [17,18]. Initial reports indicated that diagnosis of CEF during laparoscopic cholecystectomy was associated with an increased rate of conversion to laparotomy [19,20]. Currently, because of the increased proficiency of surgeons in advanced laparoscopic techniques, including intracorporeal knotting and suturing, the duodenal mobilization conversion rate is minimal, and CEF is no longer deemed a laparoscopic limitation [20,21]. In our series, four procedures commenced laparoscopically but were switched to open surgery due to technical difficulties, while three cases were started as open-only. The average duration of hospital stay in our series was approximately 13 days (ranging from eight to 18 days). Our study recorded one mortality, representing 14% (1/7), which closely aligns with the literature, indicating rates between 15% to 22% [5, 10, 18].

Limitations

The retrospective design and small sample size (n=7) limit the generalizability of our findings, as a larger cohort would yield more robust statistical conclusions. Additionally, the series of cases evaluated represents the experience of a single tertiary care center, and long-term follow-up data were not included.

## Conclusions

Preoperative diagnosis of CEFs is difficult, and intraoperative identification presents a significant challenge for surgeons, necessitating a modification of the intended treatment, usually an elective cholecystectomy. With the widespread adoption of laparoscopic cholecystectomy for symptomatic cholelithiasis, complications such as CEFs have become increasingly rare. However, they continue to be encountered in tertiary care centers, often as a consequence of neglected or long-standing cholelithiasis. Timely diagnosis and appropriate surgical management remain key to reducing morbidity associated with this uncommon but significant complication.
